# Draft Genome Sequence of *Helicobacter suis* Strain SNTW101, Isolated from a Japanese Patient with Nodular Gastritis

**DOI:** 10.1128/genomeA.00934-16

**Published:** 2016-09-08

**Authors:** Hidenori Matsui, Tetsufumi Takahashi, Somay Y. Murayama, Ikuo Uchiyama, Katsushi Yamaguchi, Shuji Shigenobu, Masato Suzuki, Emiko Rimbara, Keigo Shibayama, Anders Øverby, Masahiko Nakamura

**Affiliations:** a Department of Infection Control and Immunology, Kitasato Institute for Life Sciences and Graduate School of Infection Control Sciences, Kitasato University, Minato-ku, Tokyo, Japan; b School of Pharmaceutical Sciences, Kitasato University, Minato-ku, Tokyo, Japan; c School of Pharmacy, Nihon University, Funabashi-shi, Chiba, Japan; d Data Integration and Analysis Facility, National Institute for Basic Biology (NIBB), Myodaiji, Okazaki-shi, Aichi, Japan; e Functional Genomic Facility, NIBB, Myodaiji, Okazaki-shi, Aichi, Japan; f Department of Bacteriology II, National Institute of Infectious Diseases, Musashimurayama-shi, Tokyo, Japan

## Abstract

We present here the draft whole-genome shotgun sequence of an uncultivated strain SNTW101 of *Helicobacter suis*, which has been maintained in the stomachs of mice. This strain was originally isolated from gastric biopsy specimens of a urea breath test-negative Japanese patient suffering from nodular gastritis.

## GENOME ANNOUNCEMENT

*Helicobacter suis*, naturally colonizing the stomach of pigs, is the most prevalent non-*H. pylori Helicobacter* species in humans with gastric diseases (1–4). We isolated *H. suis* strain SNTW101 from a urea breath test (UBT)-negative Japanese woman with nodular gastritis in 2008 (5). We have since maintained *H. suis* SNTW101 in the stomachs of female C57BL/6 mice, because this strain has not yet been successfully grown *in vitro* (5). The repeated inoculations with gastric mucosal homogenates from infected mice to uninfected mice have been performed at the intervals of three to six months (5).

The genomic DNA of *H. suis* SNTW101 was prepared from the mouse gastric mucosa by treatment with anti-*H. pylori* antibody-coated magnetic beads (5), because the infected mouse stomachs contained large quantities of endogenous *Lactobacilli* in addition to *H. suis* (6). A whole-genome shotgun library was generated from 68 ng of the purified genome using a TruSeq DNA sample preparation kit (Illumina Inc., San Diego, CA) following the manufacturer’s protocol, but with four PCR cycles to reduce amplification bias. The library was sequenced in two lanes using HiSeq1500 with 151-bp paired-end readings, which yielded 255.6 million paired-end reads (77.2 Gb). After adapter trimming using Cutadapt 1.4.1 (7), the reads were mapped onto the mouse genome (GRCm38.p1) using Bowtie2 (8) to identify contaminations derived from the host mouse genome. The unmapped reads (4.7 million paired-end reads) were then assembled using Velvet 1/2/10 (9) with optimized parameters (–k 111 and −cov cutoff 12). The resulting assembly consisted of 672 contigs with a total length of 2,322,207 bp. To identify contigs derived from the *H. suis* genome, we conducted the following analyses: (i) the contigs were compared with the published draft genome sequences of *H. suis* HS1 and HS5 (10) using BLASTn (11). The contigs satisfying the bidirectional best-hit criterion or an identity of ≥90% were extracted as candidates. (ii) The contigs were searched against the NCBI-nr database using BLASTx, and whether the source organism of the best hit belonged to the genus *Helicobacter* was checked. (iii) The median read coverage of the contigs identified as *H. suis*-derived was 107×, and we eliminated those with low (<50) or high (>200) coverage. The final data set consisted of 42 contigs with a total length of 1,608,632 bp and *N*_50_ of 132,024 bp, covering the entire lengths of existing the *H. suis* genomes of HS1 (1,635,292 bp) and HS5 (1,669,960 bp) (10).

Although *H. suis* SNTW101 was isolated from a UBT-negative patient, the putative gene cluster—including *ureA* and *ureB*, which encode the structural subunits of urease—was present in the chromosome. Indeed, *in vitro* studies of gastric mucosal homogenates from infected mice displayed urease activity. The *H. suis* SNTW101 genome sequence will contribute to the understanding of this pathogen’s virulence mechanism.

### Accession number(s).

The draft whole-genome shotgun sequence of *H. suis* SNTW101 has been deposited in DDBJ/ENA/GenBank under the accession no. BDAO00000000. The version described in this paper is the first version, BDAO00000000.1.

## References

[1] Flahou B, Rimbara E, Mori S, Haesebrouck F, Shibayama K 2015 The other helicobacters. Helicobacter 20:62–67. doi:10.1111/hel.12259. 26372827

[2] Liang J, De Bruyne E, Ducatelle R, Smet A, Haesebrouck F, Flahou B 2015 Purification of *Helicobacter suis* strains from biphasic cultures by single colony isolation: influence on strain characteristics. Helicobacter 20:206–216. doi:10.1111/hel.12192. 25582323

[3] Øverby A, Yamagata Murayama S, Matsui H, Nakamura M 2016 In the aftermath of *Helicobacter pylori*: other helicobacters rising up to become the next gastric epidemic? Digestion 93:260–265. doi:10.1159/000445399. 27160882

[4] Nakamura M, Øverby A, Murayama SY, Suzuki H, Takahashi T, Takahashi S, Matsui H 2016 Gastric non-*Helicobacter pylori* Helicobacter: its significance in human gastric diseases, p. 121–140. In Suzuki H, Warren R, Marshall B (ed), Helicobacter pylori, Part II. Springer , Japan, Tokyo. doi:10.1007/978-4-431-55705-0_8.

[5] Matsui H, Takahashi T, Murayama SY, Uchiyama I, Yamaguchi K, Shigenobu S, Matsumoto T, Kawakubo M, Horiuchi K, Ota H, Osaki T, Kamiya S, Smet A, Flahou B, Ducatelle R, Haesebrouck F, Takahashi S, Nakamura S, Nakamura M 2014 Development of new PCR primers by comparative genomics for the detection of *Helicobacter suis* in gastric biopsy specimens. Helicobacter 19:260–271. doi:10.1111/hel.12127. 24673878

[6] Matsui H, Takahashi T, Øverby A, Murayama SY, Yoshida H, Yamamoto Y, Nishiyama K, Seto Y, Takahashi T, Mukai T, Nakamura M 2015 Mouse models for assessing the protective efficacy of *Lactobacillus gasseri* SBT2055 against *Helicobacter suis* infection associated with the development of gastric mucosa-associated lymphoid tissue lymphoma. Helicobacter 20:291–298. doi:10.1111/hel.12203. 25627811

[7] Martin M 2011 Cutadapt removes adapter sequences from high-throughput sequencing reads. EMBnet 17:10–12. doi:10.14806/ej.17.1.200.

[8] Langmead B, Salzberg SL 2012 Fast gapped-read alignment with Bowtie 2. Nat Methods 9:357–359. doi:10.1038/nmeth.1923. 22388286 PMC3322381

[9] Zerbino DR, Birney E 2008 Velvet: algorithms for *de novo* short read assembly using de Bruijn graphs. Genome Res 18:821–829. doi:10.1101/gr.074492.107. 18349386 PMC2336801

[10] Vermoote M, Vandekerckhove TT, Flahou B, Pasmans F, Smet A, De Groote D, Van Criekinge W, Ducatelle R, Haesebrouck F 2011 Genome sequence of *Helicobacter suis* supports its role in gastric pathology. Vet Res 42:51. doi:10.1186/1297-9716-42-51. 21414191 PMC3065412

[11] Altschul SF, Madden TL, Schäffer AA, Zhang J, Zhang Z, Miller W, Lipman DJ 1997 Gapped BLAST and PSI-BLAST: a new generation of protein database search programs. Nucleic Acids Res 25:3389–3402. doi:10.1093/nar/25.17.3389. 9254694 PMC146917

